# Eosinophil Polymorphonuclear Leukocytes in TB: What We Know so Far

**DOI:** 10.3389/fimmu.2019.02639

**Published:** 2019-11-14

**Authors:** Senbagavalli Prakash Babu, Prakash B. Narasimhan, Subash Babu

**Affiliations:** ^1^Jawaharlal Institute of Postgraduate Medical Education and Research (JIPMER), Pondicherry, India; ^2^Preventive and Social Medicine, Jawaharlal Institute of Postgraduate Medical Education and Research (JIPMER), Pondicherry, India; ^3^National Institute of Research in Tuberculosis (ICMR), Chennai, India; ^4^Laboratory of Parasitic Diseases, National Institute of Allergy and Infectious Diseases (NIH), Bethesda, MD, United States

**Keywords:** eosinophils, tuberculosis, polymorphonuclear leukocytes, granulocytes, granule proteins

## Abstract

Knowledge on the cellular immune responses to infection with *Mycobacterium tuberculosis* has improved drastically in recent years. Though eosinophils and hypereosinophilia are linked with the disease process of tuberculosis, there is paucity of information to prove the actual role played by these polymorphonuclear leukocytes. The aim of this review is to provide an overview of the existing literature on what is known so far about eosinophils and tuberculosis.

## Introduction

Eosinophilia is a classical hallmark of infectious diseases, and mycobacterial infections frequently show eosinophil recruitment in both naturally occurring human infections and in experimental animal infections. The precise function of eosinophils in the host immune responses in TB remains poorly understood. They have been noted in the lesions of human patients and animal models with mycobacterial infection, but their role has never been investigated fully. There are little data available in the literature to prove the function of eosinophils in the host protection in infection with TB and in the inflammatory pathology of the disease. The aim of this review is to provide an overview of what is known in the literature till date about polymorphonuclear eosinophils in TB.

## Cellular Immune Response In TB

Our knowledge of the cellular response to infection with *Mycobacterium tuberculosis* (MTB) infection has improved dramatically over the years. The chronicity of Mtb infection results in an inflammatory and dynamic environment within which the acquired cellular response must act. In this regard, the impact of the mycobacterium on cellular inflammation is a key element. Cellular immune responses in TB involves dendritic cells as inducers of activation of naive T cells, which is followed by the migration of activated T cells to the primary site of infection, which takes place 15–18 days post-infection (1). The next step in the protection is accumulation of activated T cells in the inflammatory lesion, activation of infected phagocytes such as macrophages, and the association of multifunctional and cytolytic antigen-specific lymphocytes. Analysis of cellular responses has shown that infected and diseased individuals express a high frequency of multifunctional T cells (2). Antigen-specific interleukin (IL)-17- and IL-22-producing functional T-cell subsets have also been identified recently in humans exposed to TB (3) and linked to enhanced pathology through increased presence of granulocytes in TB granuloma (4).

Although acquired cellular immunity is the focus of many studies, the study of the innate response of humans to infection has been gaining momentum recently. Neutrophils have been implicated in antimycobacterial immunity due to their ability to provide antibacterial activity (5). Importantly, an inverse relationship between the number of peripheral neutrophils and the risk of Mtb infection in contacts of pulmonary TB patients was observed (6). One interesting study reported an increase in Type I interferon (IFN)αβ-inducible transcripts in the blood of active TB patients compared to healthy controls, and the transcripts are found to be overexpressed in neutrophils and monocytes (7). Also, the ability of natural killer (NK) cells to lyse infected human alveolar macrophages plays an important role in the earliest response to infection (8). One of the largest populations of lymphocytes within TB lesions is likely composed of B cells, and data also suggest that B cells modulate both the inflammatory and the cytokine response in host immune response to TB (9). Despite advances, there are still a lot more to understand the exact role played by the cellular immunity in TB, which is very important to improve our knowledge of this complex disease.

## Definitions Of Eosinophils

Eosinophils comprise 1–3% of total leukocytes, and the normal percentage of eosinophils in blood varies between 0.0 and 6.0%. The normal absolute eosinophil count (AEC, obtained by multiplying the percentage of eosinophils by the white blood cell) is between 30 and 350. Mild blood eosinophilia is defined as AEC between 0.5 and 1.0 × 10^9^/L (SI units) or 0.5 and 1.0 × 10^3^ cells/microliter (conventional units) and hypereosinophilia as AEC ≥1.5 × 10^9^/L (10). Mild eosinophilia in blood is common, occurring in 3–10% of individuals with atopic disease, asthma, drug hypersensitivity, and helminth infection being the frequent causes. Blood hypereosinophilia is rare and needs a complete evaluation of the underlying cause. Tissue eosinophilia is defined as eosinophils present as >20% of all nucleated cells in a bone marrow aspirate in addition to evidence of tissue infiltration of eosinophils and extracellular deposition of eosinophil granule proteins. Human eosinophils are approximately 8 μM in diameter, have a half-life of about 8–18 h (11) in circulation, and a mean blood transit time similar to neutrophils, which is about 26 h. The majority of eosinophils (>90%) in humans reside in tissues that have substantial cellular turnover and regenerative capacity, including bone marrow, lymphoid tissues, uterus, gastrointestinal tract (except esophagus) under normal conditions and in sites of wound repair and solid tumors in case of pathology.

## Research Facts On Eosinophils

Eosinophils are good granulocyte partners of neutrophils but are less liable to be studied compared to neutrophils for several reasons: (a) Percentage of blood eosinophils is 0.0–6.0% compared to 50–60% of blood neutrophils in peripheral blood. (b) More than 90% of eosinophils reside in tissues under normal conditions, which make it hard to study and isolate them from peripheral blood. (c) There is still a lack of a single surface marker, which is uniquely expressed on the surface of eosinophils. Nonetheless, using flow cytometry, eosinophils could be gated from the granulocyte population based on cell size and granularity (forward and side scatter patterns) and surface expression of CD9, CCR3, and Siglec-8 (12). (d) As mentioned above, absence of a single surface marker uniquely present on eosinophils makes it harder to isolate them from whole blood. However, highly purified eosinophils can be obtained from peripheral blood by a combination of density gradient separation and negative selection using antibody-based magnetic negative selection protocol. This method can yield 99% pure eosinophils from both normal donors and hypereosinophilic patients. Having a prior idea of the percentage of eosinophils through differential cell counts and performing cytospin of the PBMC and granulocyte layers after density gradient centrifugation will provide additional help in eosinophil isolation and the obtained purity. Some of the practical difficulties in handling eosinophils include (i) improper isolation procedure might lead to activation of eosinophils and release of preformed granular proteins; (ii) existence of heterogeneity of eosinophils including hypodense and normodense forms; and (iii) eosinophils do not withstand freeze-thaw cycle. Nevertheless, with the advent of recent transcriptomic, proteomic, epigenomic, and immunologic research tools, the availability of eosinophil-specific mouse models and increasing numbers of eosinophil-targeted therapies in human, there has been increasing interest in understanding this unique cell in the context of human pathology.

## Eosinophils Have The Biologic Arsenal To Combat Bacterial Infection

Eosinophils possess a large number of cell-surface molecules including Toll-like receptors, adhesion molecules, chemokine, complement and chemotactic factor receptors, immunoglobulin receptors, apoptotic signaling molecules, prostaglandins, and leukotriene receptors (13). These cells have complex extracellular- and intracellular features, enabling them to respond to the changing environment, and the complexity is represented as figure and reviewed earlier (14). Eosinophils store a wide range of preformed, biologically active proteins, including cationic proteins [such as major basic protein (MBP), eosinophil peroxidase (EPO), eosinophil cationic protein (ECP), and eosinophil-derived neurotoxin (EDN)], cytokines, chemokines, and growth factors. Eosinophil granule proteins possess antibacterial, bactericidal (15), induction of oxidative damage and mutagenesis of DNA and RNA (16, 17) properties. Eosinophils exhibit degranulation through cytolytic degranulation, piecemeal degranulation (18), and as secretagogues (18, 19) in response to stimuli. Eosinophils possess terminal effector functions including direct cytotoxic effects of granule proteins, inducing antibody (20)- and complement (21)-mediated cytotoxicity, phagocytic ability (22), and expulsion of extracellular DNA traps (23). Human eosinophils possess lipid bodies that are specific inducible sites of eicosanoid mediator formation (24), which are known to control the outcome of bacterial infection (25).

## Eosinophils And TB

### Animal Models

Low-dose aerosol infection with *M. tuberculosis* showed rapid accumulation of eosinophils in broncheoalveolar lavage and lung granulomas in guinea pigs (26). Recruitment of eosinophils was found within mycobacterial granuloma in different experimental animal models. In an acute inflammatory murine model, it was observed that eosinophils are not only attracted to live mycobacteria but also phagocytosed them, and this effect was not observed with heat-killed mycobacteria (27). Eosinophils were thought to affect the susceptibility of mycobacterial infection in IFN-**γ**-deficient mouse model (28). It was observed that infection with Bacillus Calmette–Guérin (BCG) induces pleural eosinophil accumulation and activation through a TLR2-dependent CCR3 mechanism (29). Also, recruitment of eosinophils is associated with unrestricted mycobacterial growth in MTB-susceptible strains (31) and in IFN-**γ**^−/−^ mice (30, 31).

### Human Studies

Patients with non-tuberculous mycobacterial (NTM) infection had significant levels of eosinophils in peripheral blood than those infected with *M. tuberculosis*. Furthermore, patients with *M. avium*-intracellulare complex (MAI) compared to those culturing NTM other that MAI had higher eosinophil counts. Moreover, eosinophilia has been linked to prevalence of active TB in HIV-1-infected patients, suggesting a role in pathogenesis and disease susceptibility (32). On the other hand, eosinophils exhibit bactericidal potential mediated through phagocytosis, respiratory burst, and mobilization on cytotoxic proteins in the presence of bacterial infection (33, 34), which suggest a protective role of these cells in bacterial infection.

### Case Reports

The drug reaction with eosinophilia and systemic symptom (DRESS) is a drug-induced life-threatening syndrome including severe eruption, fever, hypereosinophilia, and internal organ involvement (35). DRESS caused by anti-TB drugs is rarely reported and is mostly due to rifampicin (36). A 39-year-old Cambodian woman with TB presented with DRESS syndrome with hypereosinophilia at 1,400 cells/mm^3^, which was diagnosed to be induced by ethambutol (37). Peripheral blood and pulmonary eosinophilia was evident in three pulmonary TB patients with elimination of eosinophilic inflammatory process in two of the patients with successful anti-TB treatment, and tissue pathology was mainly associated with the discharge of toxic eosinophil proteins (38, 39).

## Mechanisms Of Eosinophil-Mediated Effector Functions In TB

Despite the existence of several reports on eosinophilia and TB, their actual contribution in controlling MTB growth is unknown. However, there are several reports supporting the fact that eosinophil cationic proteins are mycobactericidal promoting lysis. In an *in vitro* study, it was observed that human EPO induced surface alteration followed by lysis of *M. tuberculosis* bacilli, and EPO-containing macrophages exhibited strong antimycobacterial activity (40).

It was also found that eosinophils could release defensins in response to BCG or cell wall components of Mycobacteria, which can directly kill BCG *in vitro* (41). With direct relevance to human TB, we found in a preliminary study that circulating immune complexes isolated from patients with TB exhibited a profound effect on overall granulocyte functions with activation of certain effector mechanisms including release of human neutrophil peptides 1–3 and dampening of others (42). In a very recent study published by Moideen et al. (43), elevated levels of MBP and EDN were observed in patients with pulmonary TB (PTB), and a decrease of eosinophil granule proteins was observed with anti-tubercular treatment (ATT). Similar results were observed by us in an ongoing study that is aimed to understand the biologic relevance of eosinophils in human MTB infection that both irradiated and live MTB induced release of eosinophil-specific granule proteins EDN and ECP (44). All of these suggest a possible role played by the eosinophil granule proteins in eosinophil-specific effector functions in TB.

In addition to the release of granular proteins, yet another appreciated function of eosinophils is their capacity to synthesize, store within intracellular granules, and very rapidly secrete a highly diverse repertoire of cytokines. The growing list includes IL-12, IFN-g, IL-4, IL-5, IL-13, RANTES, IL-8, eotaxin, GM-CSF, IL-3, TGF-α, stem cell factor, TNF-α, IL-6, IL-16, IL-2, and IL-10 (45). Eosinophils also communicate with a range of innate immune cells (such as mast cells, dendritic cells, macrophages, and neutrophils) and serve to bridge innate and adaptive immunity by regulating the production of chemokines and cytokines (CCL17, CCL22, IL-6) and *via* antigen presentation. However, there is a lack of concrete evidence, and extensive research is much needed to prove the actual modulatory roles played by eosinophils in the context of TB.

## Eosinophil-Specific Animal Models In TB

A roadblock to studying the functions of eosinophils *in vivo* is the lack of appropriate experimental animal models. One of the models available is the Δdbl GATA mice, which is the eosinophil lineage-ablated mice developed by the depletion of the high-affinity GATA-binding site in the GATA-1 promoter (46). The PHIL mice, developed using an eosinophil-specific promoter from the EPO gene to drive the expression of diphtheria toxin A, a cytocidal protein is yet another hallmark eosinophil-depleted model (47). Other strains of mice include targeted gene knockouts of IL-5, eotaxin1/2 (or its receptor CCR3), MBP-1, and EPO. There is very little to none in the literature on the use of these eosinophil-specific animal models to understand the immunology of TB. One study demonstrated that treatment with anti-IL-5 demonstrated reduced mycobacterial growth in the lung of *Mycobacterium bovis* bacillus Calmette-Guérin-infected gamma interferon receptor-deficient mice. Much more studies using these animal models are warranted in the field of TB to elaborate the role played by eosinophils.

## Helminth-TB Coinfection

Over two billion people worldwide are infected with parasites, and over one-third of the world's population is infected with MTB with a high degree of geographical overlap in the occurrence of these two disease conditions. Interestingly, the immune responses induced by the extracellular helminths (mostly Th2) and those induced by the intracellular MTB (Th1 immunity) are often mutually antagonistic and might have potential skewing of the host immune system (48). Although CD4^+^ Th2 cells are the important source of IL-4, IL-5, IL-9, IL-10, and IL-13, other cell types including eosinophils, basophils, and innate lymphoid cells (ILCs) are capable of producing these cytokines in response to helminth infections (49). In a study conducted in Ethiopia, it was observed that a high burden of intestinal parasites is observed among patients coinfected with TB, and there is a correlation of hypereosinophilia with asymptomatic helminth infection (50). Dominant Th2 response seen due to prevalence of parasitic infections in developing countries might tip the balance of Th1/Th2 immunity, increasing susceptibility to TB. Further studies on the impact of eosinophils in helminth-TB might produce interesting immunomodulatory pathways.

## Conclusions

The exact role of eosinophils in the host immune response to mycobacterial infection in experimental and clinical TB remains to be established. Prospective and translational research could identify the causation and so determine whether our finding may be utilized within future management strategies of TB and in coinfections. In light of these results, further investigation into the impact of Th2 immune responses on clinical mycobacterial disease is warranted. Extensive analysis of TB-associated eosinophilia and correlation with disease outcome would represent a further goal to better understand the role of eosinophils during TB infection and progression of the disease.

## Author Contributions

SP involved in drafting the review. PN and SB are involved in revising the review critically for important intellectual content.

### Conflict of Interest

The authors declare that the research was conducted in the absence of any commercial or financial relationships that could be construed as a potential conflict of interest.
